# Long-Term Effect of Switching From an Anti-CGRP Receptor to an Anti-CGRP Ligand Antibody in Treatment-Refractory Chronic Migraine: A Prospective Real-World Analysis

**DOI:** 10.1007/s13311-023-01394-0

**Published:** 2023-07-10

**Authors:** Giorgio Lambru, Valeria Caponnetto, Bethany Hill, Susanna Ratti, Simona Sacco, Madeleine Murphy, Jessica Briscoe, Anna P. Andreou

**Affiliations:** 1 The Headache and Facial Pain Service, Guy’s and St Thomas’ NHS Foundation Trust, London, UK; 2 Wolfson CARD, Institute of Psychiatry, Psychology and Neuroscience, King’s College London, United Kingdom, London, UK; 3 Headache Research‑Wolfson CARD, Institute of Psychiatry, Psychology and Neuroscience, King’s College London, London, UK; 4 Department of Applied Clinical Sciences and Biotechnology, University of L’Aquila, L’Aquila, Italy

**Keywords:** Erenumab, Fremanezumab, Calcitonin gene-related peptide, Chronic migraine, Refractory migraine

## Abstract

In migraine patients with a poor response to a calcitonin gene-related peptide monoclonal antibody against the receptor, switching to a calcitonin gene-related peptide monoclonal antibodies against the ligand may be beneficial. This was a long-term real-world prospective analysis conducted in treatment-refractory chronic migraine patients coming from two large tertiary referral headache centres, who did not achieve a meaningful response to erenumab and were switched to fremanezumab. Responders to fremanezumab were considered those who achieved at least 30% reduction in monthly migraine days by month 3, compared to the post-erenumab baseline. Secondary efficacy and disability outcomes were analysed. Thirty-nine patients (female *n* = 32, 82.1%; median age: 49 years old, IQR = 29.0–56.0) were included. After three months of treatment with fremanezumab, ten out of 39 patients (25.6%) were considered responders. Four of the 11 patients who continued fremanezumab became responders at month 6, increasing the number of responders to 14 patients (35.9%). Responders received a median of 12 injections (IQR = 9.0–18.0) at the time of the analysis. After the last treatment, 13 patients (33.3%) remained responders. The number of mean monthly migraine days significantly decreased from 21.4 at baseline (IQR = 10.7–30.0) to 8.6 (IQR = 3.8–13.9) at the last follow-up. Painkillers intake and HIT-6 score were significantly reduced at the last follow-up. About 1/3 of patients with treatment refractory chronic migraine who have a disappointing response to erenumab and switch to fremanezumab, obtained a meaningful and sustained improvement of their migraine load over time, supporting the appropriateness of this therapeutic approach in clinical practice.

## Introduction

Migraine, especially its chronic variant, is a relevant epidemiological and public health matter, especially considering aspects related to its prevalence [1], comorbidities [2, 3] and impact on patients’ life [4]. Abortive and preventive treatments are directed at reducing the number and/or intensity of the attacks and related disability. The pharmacological preventive treatment of migraine involves different drug classes, including b-blockers, antidepressants, calcium channel blockers, antiepileptics, onabotulinum toxin type A for chronic migraine (CM) and more lately the monoclonal antibodies (mAbs) against calcitonin gene-related peptide (CGRP) or its receptor. Two CGRP mAbs against the ligand, fremanezumab and galcanezumab, and one CGRP mAb against the receptor, erenumab, have been approved for migraine prevention in the United Kingdom (UK) and Europe. Although generally very effective in migraine prevention, up to 60% of CM and difficult-to-treat CM patients treated with anti-CGRP mAbs do not obtain a meaningful improvement [5]. These findings are slightly more promising when coming from the real-world data [6, 7], though the sustained long-term relief in treatment-resistant CM patient seem to be poor [8, 9]. The group of patients non-responding/tolerating preventive treatments including the CGRP mAbs are considered treatment refractory based on a recent consensus statement. The definition of refractory migraine includes failure to respond/tolerate the established preventive treatments including onabotulinum toxin type A and a CGRP mAb [10]. Nonetheless, in patients with minimal or no response to a CGRP mAb against the receptor, it may be reasonable to switch to a CGRP mAb against the ligand, in light of their slightly different mode of action. Preliminary retrospective data coming from a small series has suggested that between 1/3 to half of patients who failed to respond to erenumab may respond to galcanezumab or fremanezumab [11].

Understanding the effectiveness of a CGRP mAb against ligand in patients not responding sufficiently to a CGRP mAb against the receptor may increase the treatment opportunities of these highly disabled patients and improve the understanding of the effect of modulating the CGRP at different levels of its pathways in migraine. For these reasons, we prospectively evaluated the short- and long-term effectiveness of fremanezumab in a difficult-to-treat population of CM patients who previously failed to respond to erenumab.

## Methods

This is a prospective clinical analysis conducted in two large tertiary headache referral centres: the Headache and Facial Pain Service at Guy’s and St Thomas’ (GSTT) National Health System (NHS) Foundation Trust of London (UK) and the Headache Centre of Avezzano-L’Aquila (Italy). Patients included were treated with erenumab between November 2018 and January 2020 and were followed up until June 2022. The guidelines for prescription, assessment and discontinuation differ in the UK and Italy and are summarised in Table 1. MIDAS (Migraine Disability Assessment) questionnaire changes from baseline was not analysed given that it was not used in the UK patients. The treatment pathway for this analysis consisted of a baseline of 4-week pre-treatment with erenumab, followed by at least 3-month treatment period with erenumab, a variable treatment break and finally at least a 3-month treatment period with fremanezumab. A month was defined as 30 calendar days.

**Table 1:** Criteria for mAbs prescription and discontinuation in the United Kingdom and Italy

	**United Kingdom**	**Italy**
**Diagnosis**	Episodic or chronic migraine with or without aura	Episodic or chronic migraine with or without aura
**Minimum headache monthly frequency**	≥ 4 days/month	≥ 8 days/month
**Previous treatment failed**	At least 3 of any migraine preventatives	At least 3 treatments including an antiepileptic, a tricyclic antidepressant and a beta-blocker. For chronic migraine one of the three mandatory drugs may be onabotulinum toxin A
**Other criteria**	Consider contraindications as per drug licence	- Consider contraindications as per drug licence - Migraine Disability Assessment (MIDAS) score ≥ 11
**Criteria for treatment continuation**	After 3 months of treatment: - 50% reduction of monthly migraine days in episodic migraine compared to baseline - 30% reduction of monthly migraine days in chronic migraine compared to baseline	After 3 months and 6 months of treatment: - 50% reduction of MIDAS score compared to baseline
**Treatment discontinuation/pausing**	- Not mandatory	- Mandatory after 12 (for fremanezumab and galcanezumab) or 13 injections (for erenumab)
**Restarting criteria**	- Patient has to fulfil baseline criteria	- Patient has to fulfil again baseline criteria

*MIDAS* Migraine Disability Assessment

### Participants

Adult patients meeting the International Headache Society (IHS) criteria for CM and receiving treatment with fremanezumab after having been treated with erenumab were included in the analysis [12]. As per national reimbursement criteria both in the UK and Italy, all patients had to fail at least three preventive treatments before receiving erenumab. These treatments belonged to the following classes: beta-blockers (propranolol, atenolol), tricyclics (amitriptyline and nortriptyline), anticonvulsants (topiramate, gabapentin, pregabalin and sodium valproate), angiotensin II receptor blocker (candesartan), botulinum toxin type A (BoNT/A), greater occipital nerve blocks (GONBs) calcium channels blockers (flunarizine), serotonin antagonists (pizotifen), serotonin and norepinephrine reuptake inhibitors (SNRI), namely, venlafaxine and duloxetine, other antidepressants (mirtazapine) and noninvasive neuromodulation therapies (single-pulse transcranial magnetic stimulation). The latter treatment is not available in Italy. Consecutive patients treated with erenumab (70 mg or 140 mg) for at least three months who either did not respond, or obtained a minimal but not meaningful enough benefit, or who initially responded but in whom the effectiveness wore off over time, were included in the analysis. These patients were switched to fremanezumab 225 mg/month after a variable interval period. We assessed the short- and long-term response to fremanezumab to establish the sustained effectiveness in responders.

### Outcome Measures and Timepoints

A migraine-specific diary and the Headache Impact Test-6 (HIT-6) score were used to capture efficacy and disability measures. Patients were required to fill in the headache diary on a daily basis along with HIT-6 scores every month for the duration of the treatment period and while switching between the two CGRP mAbs. Data were entered in an electronic macro database for analysis. Collected variables included patients’ age, sex, diagnosis, comorbidities, years with CM, presence of aura, number of preventatives failed before first erenumab administration and other preventives failed between last erenumab administration and first fremanezumab administration. Efficacy outcomes collected included monthly migraine days (MMDs), monthly headache days (MHDs), monthly crystal clear headache free days, monthly abortive treatment days, 3-month HIT-6 score and changes in concomitant preventatives. These outcomes were collected during the last month before the first fremanezumab administration (baseline) and at the third and sixth treatment with fremanezumab. Moreover, the treatment interval between last erenumab and first fremanezumab injection, the total number of injections received for both CGRP mAbs and the side effects during fremanezumab treatment were documented. Data was collected during patients’ clinical assessment at baseline and at follow-up appointments every three months during treatment periods.

The main efficacy outcome was changes from baseline in the mean MMDs at month 3. The cut-off outcome for treatment continuation was reduction in the mean MMD of at least 30% after three monthly fremanezumab injections, compared to the post-erenumab baseline. These patients were considered as responders to fremanezumab. Secondary outcomes included: reduction of ≥ 50% and ≥ 75% in MMDs at month 3 and 6 compared to baseline, changes in mean MHDs, changes in mean monthly crystal clear days, changes in mean monthly abortive treatment days and 3-month Headache Impact Test-6 (HIT-6) at each timepoint. Moreover, side effects at each timepoint were also documented to evaluate safety and tolerability.

### Statistical Analyses

Patients’ characteristics were reported with descriptive statistics. Moreover, all continuous outcomes were summarised using median and interquartile range (IQR) and compared with baseline values utilising non-parametric analysis and the Wilcoxon rank test for paired samples; all categorical outcomes were expressed as counts and percentages. Power analysis for sample calculation was not performed since this was an observation of clinical practice. All statistics were performed with SPSS version 21.0 (IBM Corp., Armonk, NY, USA) with an accepted statistical error ≤ 0.05. Patients with missing data were excluded from analyses related to the affected variable; for variables reporting more than 10% of missing data [13], we only performed descriptive statistics.

### Ethics

Audit under current national guidelines in UK does not require research ethics committee review (http:// www. hra-decisiontools.org.uk/research/). Italian patients were already included in a real-life study on CGRP mAbs, which was approved by the local Ethical Committee of the University of L’Aquila. Patients provided informed consent.

## Results

### Patients’ Characteristics

Overall, 39 CM patients were included in this analysis, 33 from the GSTT Headache Service, UK, and six from the Avezzano-L’Aquila Headache Centre, Italy. Patients had a median age of 49.0 years (IQR = 29.0–56.0), and they were mainly female (*n* = 32, 82.1%). At the time of the analysis, patients had experienced CM for a median of 10.0 years (IQR = 5.0–17.0). Migraine with aura was diagnosed in 13 (33.3%) of patients. Before starting erenumab, patients had failed a median of 7.5 (IQR = 6.0–11.0) migraine preventatives; onabotulinum toxin A failure was reported in 35 (89.7%) of patients (Table 2).

**Table 2:** Patients’ demographic and clinical characteristics (*N* = 39)

**Variables**	
Age in years, median (IQR)	49.0 (29.0–56.0)
Females, *N* (%)	32 (82.1%)
Comorbidities	*N*
None	5
*Mental health disorders:*	*11*
Anxiety and depression	7
Generalised anxiety	1
Post-traumatic stress disorder	1
Major depression	2
Chronic non-headache pain	8
Sleep disorders	4
Irritable bowel syndrome	3
Asthma	3
Raynaud’s syndrome	2
Others	5
Years with chronic migraine^a^, median (IQR)	10.0 (5.0–17.0)
Aura, *N* (%)	13 (33.3%)
Number of preventatives failed before fremanezumab (excluding erenumab), median (IQR)	7.5 (6.0–11.0)
Failed onabotulinum toxin A, *N* (%)	35 (89.7%)
Tried and failed other preventatives between erenumab and fremanezumab, *N* (%)	14 (36.8%)
Number of failed preventatives between erenumab and fremanezumab, median (IQR)	2.0 (1.0–3.0)

^a^Missing data (*N* = 5)

### Primary Outcomes in Patients Switching to Fremanezumab

**Fig. 1:**
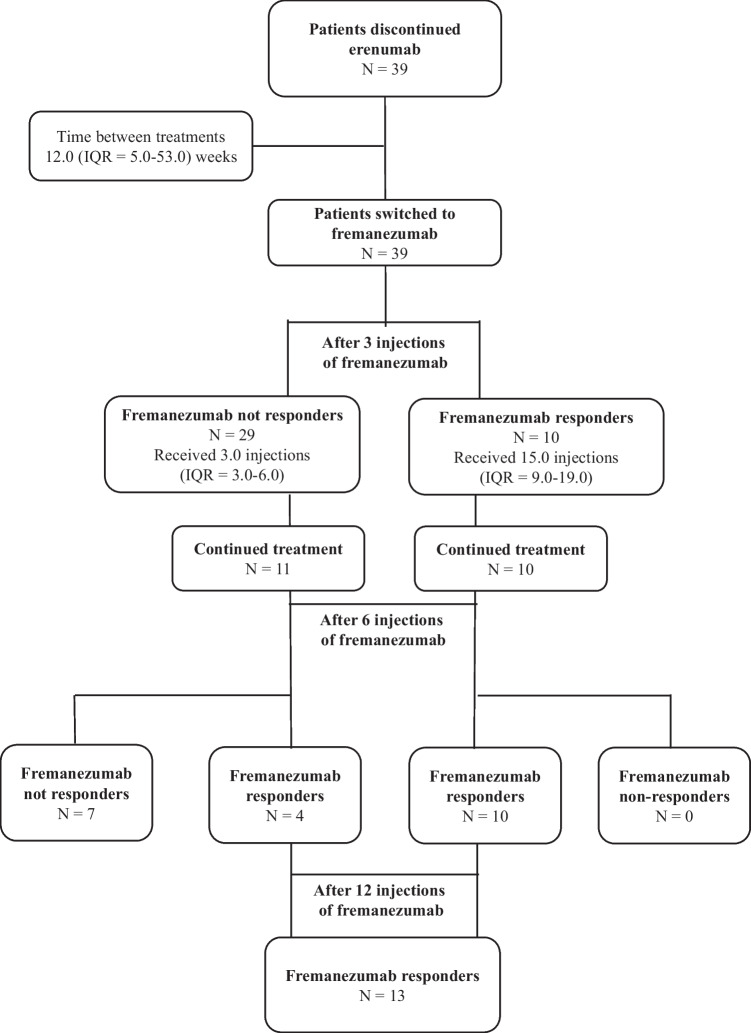
Patients’ pathway and response to fremanezumab after switching from erenumab. Number of injections and time between treatments are expressed in median

All baseline headache characteristics are outlined in Table 3. At the time of treatment discontinuation, patients had received a median of 13.0 (IQR = 7.0–21.0) erenumab injections. The choice of continuing erenumab over 3–6 months in some patients with only minimal benefit was dictated by the lack of any alternative treatment options at that stage. After discontinuing erenumab, patients received the first dose of fremanezumab after a median of 12.0 (IQR = 5.0–53.0) weeks. The mean MMD at the month before starting fremanezumab was 21.4 (10.7–30.0). After three months of treatment with fremanezumab, 29 patients (74.4%) were not responders, whereas ten patients (25.6%) obtained at least a 30% reduction in MMD, hence were considered responders. Among these ten patients, six reported a ≥ 50% reduction in MMD and one of them reported a 75% reduction in MMD. Response was sustained at month 6 in all ten patients. Of the non-responders at month 3, 18 patients discontinued the treatment (one patient discontinued also due to side effects), while eleven patients continued until month 6. Of those, four (36.4%) became responders at month 6. Hence, after six months treatment with fremanezumab, a total of 14 patients (35.9%) responded to fremanezumab. At this time point, ten out of 14 patients (71.4%) reported a ≥ 50% reduction in MMD and two of them reported a 75% reduction in MMD. All fremanezumab responders continued the treatment and received a median of 12 injections (IQR = 9.0–18.0) at the time of the analysis. After the last treatment, 13 patients (33.3%) remained responders (Fig. 1). About half of patients were using at least one concomitant migraine preventative before starting fremanezumab. This percentage remained stable at the last follow-up (Table 4). Patients who failed both erenumab and fremanezumab were placed on further treatments, including galcanezumab, lamotrigine, pregabalin, single-pulse transcranial magnetic stimulation, topiramate or onabotulinum toxin type A.

**Table 3:** Secondary efficacy outcomes

	**Baseline (post-erenumab)** (***N* = 39)**	**3rd month** (***N* = 39**)	***p* value**	**6th month** (***N* = 21**)	***p* value**	**Last treatment** (***N* = 16**)	***p* value**
Total monthly headache days, median (IQR)	30.0 (17.1–30.0)	25.0 (12.9–30.0)^a^ (2 md)	0.001	17.1 (6.4–23.6)	0.003	8.6 (4.3–17.1)	0.001
Monthly migraine days, median (IQR)	21.4 (10.7–30.0)	15.0 (8.6–30.0)	0.007	8.6 (5.4–19.3)	0.007	8.6 (3.8–13.9)	0.001
Crystal clear days, median (IQR)	0.0 (0.0–10.7)	1.0 (0.0–15.0)^a^ (1 md)	0.006	5.4 (0.0–20.4)	0.023	7.5 (0.0–22.8)	0.026
Monthly painkillers intake, median (IQR)	8.6 (5.4–19.3)^a^ (2 md)	0.0 (0.0–7.5)^a^ (1 md)	≤ 0.001	9.6 (5.4–13.9)	0.201	5.4 (3.6–9.6)^a^ (2 md)	0.033
HIT-6 score, median (IQR)	66.0 (63.5–70.0)^a^ (5 md)	68.5 (65.0–70.0)^a^ (15 md)	-	66.0 (64.0–71.0)^a^ (12 md)	-	62.0 (50.0–66.0)^a^ (3 md)	-

*IQR* interquartile range, *HIT-6* Headache Impact Test-6, *N* number

^a^*md* missing data

**Table 4:** Concomitant preventatives during treatment with fremanezumab

		**Baseline (*N* = 39)**	**3rd month (*N* = 39)**	**6th month (*N* = 21)**	**Last treatment (*N* = 16)**
Patients using concomitant preventatives		20 (55.5%)	21 (56.8%)	10 (50.0%)	8 (50.0%)
*N* (%)^a^		(3 md)	(2 md)	(1 md)	(1 md)
Number of concomitant preventatives, median (IQR)		1.5 (1.0–2.0)	1.0 (1.0–2.0)	1.0 (1.0–2.0)	2.0 (1.5–2.0)
	Angiotensin II receptor blockers, *n* (%)	3	4	1	2
	Antiemetics, *n* (%)	1	1	1	0
	Antiepileptics, *n* (%)	6	7	3	3
	Baclofen, *n* (%)	1	1	0	0
	Beta-blockers, *n* (%)	1	0	0	0
Type pf treatment	Onabotulinum toxin A, *n* (%)	2	2	1	1
	Calcium channel blocker, *n* (%)	2	2	1	1
	External trigeminal nerve stimulator, *n* (%)	1	1	0	0
	Lithium, *n* (%)	1	1	0	0
	Nerve blocks, *n* (%)	2	1	1	1
	Pizotifen, *n* (%)	1	1	0	0
	SSRI/SNRI, *n* (%)	6	7	2	2
	Tricyclic antidepressants, *n* (%)	4	5	2	2

*IQR* interquartile range, *N* number, *SNRI* serotonin–norepinephrine inhibitors, *SSRI* selective serotonin reuptake inhibitors

^a^*md* missing data

### Secondary Outcomes in Patients Switching to Fremanezumab

The total number of mean MMD significantly decreased from 21.4 at baseline (IQR = 10.7–30.0) to 15.0 (IQR = 8.6–30.0) at month 3 (*p* = 0.007), to 8.6 (IQR = 5.4–19.3) at month 6 (*p* = 0.007) and to 8.6 (IQR = 3.8–13.9) at the last follow-up. Treatment with fremanezumab also increased the median number of crystal clear days from 0.0 (IQR = 0.0–10.7) days at baseline to 1.0 (IQR = 0.0–15.0) at month 3 (*p* = 0.006), 5.4 (IQR = 0.0–20.4) at month 6 (*p* = 0.023) and 7.5 (IQR = 0.0–22.8) at the last follow-up. Painkillers intake and HIT-6 score were significantly reduced at the last follow-up (Table 3).

### Safety and Tolerability

Fremanezumab treatment-related side effects were reported in 20.5% of patients at month 3, 19.0% of patients at month 6 and in 18.8% of patients after the last treatment we recorded. Side effects were considered generally rated as mild and they are detailed in Table 5. One patient discontinued the treatment after the third injection due injection site reaction.

**Table 5:** Side effects with fremanezumab

	**3rd month** **(*N* = 39)**	**6th month** **(*N* = 21)**	**Last treatment** **(*N* = 16)**
Any, *N* (%)	8 (20.5%)	4 (19.0%)	3 (18.8%)
Injection site reaction, *N* (%)	2 (5.1%)	2 (9.5%)	1 (6.3)
Constipation, *N* (%)	1 (2.6%)	–	1 (6.3%)
*Others*			–
Insomnia, *N* (%)	–	1 (4.8%)	1 (6.3%)
Nightmares, *N* (%)	–	1 (4.8%)	–
Dizziness, *N* (%)	1 (2.6%)	–	–
Worsening of the headache, *N* (%)	1 (2.6%)	–	–
Nausea, *N* (%)	1 (2.6%)	–	–
Worsening of Raynaud’s syndrome, *N* (%)	1 (2.6%)	–	–
Fatigue, *N* (%)	1 (2.6%)	–	–

*N* number

## Discussion

This is the first prospective real-world analysis aiming to clarify the long-term effectiveness of a CGRP mAb-fremanezumab, against the ligand in the treatment refractory CM patients with a poor/no clinical response to a CGRP mAb against the receptor–erenumab. Since the widespread use of this novel class of migraine-specific preventive treatments in clinical practice, the choice on whether to try a second anti-CGRP pathway mAb in patients with poor/lack of response to the first anti-CGRP pathway mAb has become relevant. Our data indicates that about 1/3 of refractory CM patients who did not obtain a satisfactory response to erenumab respond to long-term exposure to fremanezumab, displaying a degree of improvement which is meaningful in the majority of responders (at least 50% reduction in mean MMDs).

Our findings are in keeping with the ones of a recently published small retrospective series of treatment-refractory CM patients who failed to respond to a 3-month trial of erenumab; in that study, about 1/3 of these patients responded to a short-term treatment with fremanezumab or galcanezumab [11]. Both patients groups were treatment refractory as per EHF consensus statement, given that they failed several preventive treatments including onabotulinum toxin A and one CGRP mAb [10]. The definition of refractory CM has evolved over time from a treatment failure threshold of two preventive treatments [14, 15] to the most recent consensus that requires the failure of all the available preventive treatments in patients with a disabling condition [10]. However it is unclear whether failure of one CGRP mAb is sufficient to label a patient as refractory. Moreover, a 3-month long treatment trial to establish the effectiveness of a CGRP mAb in the difficult-to-treat migraine population, may not be long enough. It is noteworthy that real-world data on erenumab in difficult-to-treat migraine suggested that a percentage as high as 13.5% with no response to three months of treatment, obtained between 30 and 50% response between the fourth to the sixth dose, suggesting that perseverance with treatment might be beneficial in the most complex patients [7, 16]. Most of our patients were exposed to erenumab and subsequently to fremanezumab for longer than three months for this very reason. In our fremanezumab patient group, about 1/3 of patients, who continued the treatment for six months, became responders, suggesting that the difficult-to-treat migraine population may need a 6-month trials of CGRP mAbs before their efficacy is established.

Our primary clinical outcome for treatment continuation was at least 30% reduction in mean MMD as per NICE (National Institute for Health and Care Excellence) guidelines on CGRP mAbs therapies in CM in the United Kingdom [17, 18]. However, all the RCTs with anti-CGRP mAbs in migraine patients with prior therapeutic failures (Liberty, erenumab; Conquer, galcanezumab; Focus, fremanezumab; Deliver, eptinezumab) based their primary efficacy outcome upon at least 50% response rate [19–22]. In view of the spontaneous clinical fluctuation typical of migraine including its chronic subtype [23], it is arguable that a 30% only response rate may not represent a meaningful and specific enough response, but perhaps only the natural improvement of the condition. However the CM patients included in this analysis had the condition for long time and were under our care for many years. During this time they did not show any spontaneous fluctuation of the migraine pattern from chronic to episodic. Furthermore, these patients were not just difficult-to-treat CM patients but indeed treatment-refractory, having failed 6–11 preventive treatments, besides erenumab and almost always onabotulinum toxin A. On the basis of patients characteristics, the NICE criteria and the recommendation from the chronic pain clinical trials consensus [24], it is reasonable to assume that 30% reduction in MMD in these subgroup of patients reflect a biological effect of fremanezumab.

Interestingly, the majority of the sustianed responders to fremanezumab in this analysis obtain at least 50% response, supporting the favourable effect of switcihng CGRP antibodies.

There is no published evidence on the sustained long-term effectiveness of a second CGRP mAb in patients who had already failed one. However, real-world data on treatment-resistant CM treated with erenumab showed that only a small proportion of responders, maintained their improvement long-term [8, 9]. On the contrary, our findings showed that treatment with fremanezumab demonstrated long-term sustained effectivenss in reponders (up to 18 months follow-up). Furthermore, the vast majority of long-term responders to fremanezumab obtained at least a 50% reduction in MMDs, which is a meaningful degree of improvement considering the refractory nature of their condition. Together with the German data, our findings support the appropriateness of trying a second CGRP mAb with a different mechanism of action in patients who fail to respond to erenumab.

Understanding the effectiveness of a CGRP mAb against ligand therapy in patients not responding sufficiently to a CGRP mAb against the receptor may increase the treatment opportunities of these highly disabled patients and may expand the understanding of the effect of modulating the CGRP at different levels of its pathways in migraine. Indeed, CGPR has high affinity for its receptor activity-modifying protein-1 (RAMP-1)/calcitonin-receptor like (CRL) receptor, but it also has affinity for other calcitonin family receptors including the amylin and adrenomedullin receptors [25]. Preliminary evidence has shown that interictal plasma amylin levels are higher in patients with CM [26]. Furthermore, an amylin analogue, Pramlintide, infused in migraine patients led to a migraine episode in a percentage of patients similar to that in whom CGRP was infused, suggesting a role of the amylin receptors in migraine pathogenesis [27]. Although the extent of the involvement of the amylin pathway modulation in migraine in unknown, and other mechanisms may play a role, this initial evidence may constitute a mechanistic substrate for trying CGRP mAb blocking the ligand in patients who fail to respond to the CGRP mAb blocking the receptor.

Polytherapy in migraine prophylaxis is sometimes required in the difficult-to-treat patients [28]. There is initial promising experience on the synergistic effectiveness of injectables in refractory patients, namely the combination of onabotulinum toxin A and CGRP mAb therapy compared to one of them used in monotherapy [29, 30]. About half of our patients received concomitant preventive treatments while on the CGRP mAb treatments. The percentages remained relatively stable during the course of treatment. While, in some of our cases, polytherapy was meant to help reducing the migraine load, in other cases, it was prescribed to address patients’ comorbidities as well as trying to prevent migraine symptoms. The concomitant treatments were kept stable or seldom discontinued during the treatment period, likely not affecting the interpretation of the results.

The strengths of this analysis include its prospective nature; the use of objective data collection measures; the options of a longer than 3-months erenumab trial, which allowed us to avoid that some of the fremanezumab responders were in fact delayed erenumab responders; the long-term follow-up of patients on fremanezumab, which allowed to establish the sustained effectiveness in responders to a 3-month treatment. Limitations include the lack of a control group, which cannot exclude that the fremanezumab response is driven by a placebo effect. However, the generally poor response to several preventive treatments, along with the sustained long-term effectiveness to fremanezumab, seems to suggest a biological effect of the treatment rather than simply a placebo effect. Furthermore, RCTs testing CGRP mAbs in difficult-to-treat migraine population suggest that the more preventive treatments they have failed, the lower the placebo effect is [19–22]. Our patients had failed several migraine preventive treatments by the time the tried erenumab hence a small placebo effect if any in this group was expected. It could also be argued that a 30% (and not 50%) response rate would in fact represent a natural fluctuation of migraine over time, rather than a specific biological effect of fremanezumab. However, our patients had never reported a favourable spontaneous fluctuation of their migraine while under our care, nor obtained a sustained long-term improvement with any other preventive treatments before. Taken together, it is likely that the 30% response rate was a reasonable outcome measure to assess the effectiveness of CGRP mAbs for this group of patients.

Being a treatment refractory population, it is possible that the effect of switching antibodies was lower than expected in a less refractory population. However, our group of patients reflect real-world complex CM patients treated in tertiary headache clinics. Most of our patients had a treatment break from erenumab lasting an average of three months. It might be possible that the fremanezumab improvement in some responders was simply a result of an accumulation of long exposure to CGRP mAbs, initially erenumab and then fremanezumab. However, given that patients did not experience a significant response to erenumab prescribed for an average of over one year, we assume that the fremanezumab effect was not impacted by the former treatment. Finally, patients with and without medication overuse headache were not analysed separately in this report. It may be possible that these subgroups behave differently to CGRP mAbs therapy switch over. However, given that randomised–controlled evidence have confirmed that all CGRP mAbs display similar efficacy in patients with and without medication overuse across the different migraine subtypes [31], we are confident that the lack of this subgroup analysis did not bias the final study outcome.

## Conclusions

Our prospective analysis of 39 refractory-CM patients indicates that about 1/3 of patients who do not respond to a CGRP receptor mAb (erenumab) may respond to a CGRP mAb (fremanezumab). The response to fremanezumab was substantially meaningful in the majority of responders and sustained long term. Switching from a CGRP mAb again the receptor to a CGRP mAb against the ligand may be an effective treatment strategy in refractory CM. Our data supports the benefit of longer than three months trials of CGRP mAb therapies (six months) to establish response to treatment, in view of the presence of a significant minority of delayed responders. This subgroup of delay responders are important to identify and treat, given the severe disability and paucity of treatment options for the treatment-refractory CM population.

Given the small sample size and the real-world nature of this analysis, it is possible that the beneficial effect of fremanezumab in responders may have been in part enhanced by the polytherapy approach we adopted for some patients. Large controlled studies are needed to confirm our initial findings and to elucidate weather in a less refractory population, a higher percentage of patients would respond to a switch over between CGRP mAbs.

## Data Availability

Data may be available upon appropriate request to the Corresponding Author.
